# Norwegian music students’ perceptions and experiences of challenges and resources for health

**DOI:** 10.3389/fpsyg.2023.1199423

**Published:** 2023-08-21

**Authors:** Grete Ege, Fungisai Puleng Gwanzura Ottemöller, Bente Frisk

**Affiliations:** ^1^Department of Physiotherapy, Haukeland University Hospital, Bergen, Norway; ^2^Faculty of Psychology, Department of Health Promotion and Development, University of Bergen, Bergen, Norway; ^3^Department of Health and Functioning, Western Norway University of Applied Sciences, Bergen, Norway

**Keywords:** music students, health literacy, health promotion, health psychology, salutogenesis, focus group

## Abstract

**Introduction:**

Music students in higher education experience health-related challenges linked to practice and performance, while an understanding of these challenges and access to resources to deal with them are required to promote the students´ health. Health literacy and health education are integral parts of health promotion and resources for health, which encompasses health-related knowledge and competence aiming to improve health. The aim of this study was to explore Norwegian music students’ perceptions and experiences of resources and challenges for health and address the following research question: What health-related challenges do music students in higher music education meet, and what health promoting resources do they need and use to deal with these challenges and promote their health?

**Methods:**

We conducted a qualitative study including three focus group interviews with 13 music students aged between 19 and 31 years studying classical, folk, jazz or rhythmic genres from five different music departments in Norwegian universities. The Salutogenic model of health was used as theoretical framework and a few questions regarding the concept of health literacy were included in the interviews. We used thematic network analysis to analyze the data.

**Results:**

Main health challenges were related to performance pressure and difficulties implementing good health habits in the students` daily lives. Furthermore, the findings revealed several resources that promoted the students’ health: (1) Personal resources included situational understanding, using adequate coping strategies, high motivation and participating in regular physical activity. (2) Social resources involved an understanding of the importance of social support from peers and teachers and synergy created between themselves and the audience through sharing of music. (3) Environmental resources were linked to access to good rehearsal rooms. The music students expressed a need for increased competence in health promoting routines during practice and performance and suggested that health-related topics should be an integrated part of education.

## 1. Introduction

Music students in higher education acquire high levels of skills after many years of extensive practice, often from a very young age (Philippe et al., 2019). As students and later as professional musicians, they practice intensively in order to be able to perform at a professional level (Paarup et al., 2011; Wilson et al., 2014; Porter et al., 2018; Alessandri et al., 2020). Even though performing music at high professional level can be rewarding, research has shown that musicians and music students are at risk of physical and mental health problems (Araújo et al., 2017). Previous studies found that musculoskeletal disorders related to playing a musical instrument were present in 41–93% of professional musicians and music students (Kok et al., 2016) and that 12% of professional classical musicians ‘careers ended prematurely, due to these disorders (Kaufman-Cohen and Ratzon, 2011). Moreover, musicians and music students have also been reported to experience high incidences of mental health problems like anxiety and depression, where performance anxiety (stage fright) during concerts is particularly challenging for those under the age of 30 (Kenny et al., 2014; Vaag et al., 2016, 2021). Music students have also reported that being a new student in higher music education is challenging (Pecen et al., 2018; Kegelaers et al., 2021) and they seem to report more psychological challenges than professional musicians (Kegelaers et al., 2021). Negative and unsupportive teachers in addition to competitive environments with little social support have been cited as risk factors (Pecen et al., 2018). Although previous research has demonstrated challenges that may affect music students’ health, and some strategies to promote the health of musicians have been implemented, the mental and physical health challenges persist (Kenny et al., 2014; Vaag et al., 2016, 2021; Araújo et al., 2017; Pecen et al., 2018; Kegelaers et al., 2021; Guptill et al., 2022). Matei et al. (2018) and Cruder et al. (2019) highlighted this omission and the need for health education specifically targeted at music students.

Health promotion is defined as “The process that enables people to increase control over and improve their health” (WHO, 1986). In order to be able to take control and improve health, people need to understand what builds health and be able to access and use the resources they need. Health literacy and health education are thus integral parts of health promotion (WHO, 1998). Health literacy can be simply defined as ‘the capacity to acquire, understand and use information in ways which promote and maintain good health’ (Nutbeam, 2009). Thus, health literacy can be understood as a resource for health; it is multidimensional, and has a systemic perspective (Ringsberg et al., 2018). The Norwegian strategy for health literacy emphasizes that promoting health literacy needs to be both content and context specific (HOD, 2019). In 2018, an international group of researchers developed a new health literacy tool for musicians (MHL-Q19). The tool aims to examine the relationship between physical and mental performance related health issues (PMHI), and health literacy and to determine how best to decrease PMHI (Guptill et al., 2022). ‘The occupational health literacy tool aimed to measure musicians’ abilities to access, understand, appraise and apply health information concerning their performance health’ (Guptill et al., 2022). A study to evaluate the validity and reliability of the tool applied a theoretical framework that outlines the four dimensions of health literacy (accessing, understanding, appraising and applying health information) and how they interact with the three domains of health–health care, disease prevention and health promotion (Guptill et al., 2022, p. 2). The authors concluded that the instrument provided promising evidence that music student’s health literacy is a distinct construct and that there is a need for future research to not only strengthen the tool and but also researchers’ understanding of musicians’ health literacy (Guptill et al., 2022). Given that the tool measures PMHI and health literacy quantitatively, it is important to conduct qualitative research to gain more in depth understanding of the construct. Green et al. (2019) define health education as “Any planned activity designed to produce health-or illness-related learning.” Accordingly, effective health education may result in the development of cognitive capabilities such as acquisition of information, understanding and insights and health related psychomotor and social interaction skills. Thus, health education provides opportunities to learn, health literacy is the ability to understand and apply what one has learnt and thus enable promotion of health: both health literacy and health education may therefore be considered as resources for health.

Some researchers have noted the tendency of studies on musicians’ health to focus more on challenges rather than on how to promote musicians’ health. They have called for more health promotion related topics in music education, and pointed out that musicians themselves should increase their commitment to health (Araújo et al., 2017). Several international studies have explored the health literacy and health education of music students (Ackermann et al., 2014; Baadjou et al., 2014; Chan and Ackermann, 2014; Matei et al., 2018; Baadjou et al., 2020; Matei and Ginsborg, 2022). Their findings indicated a need to target effective ways to meet musicians physical, psychological and social health risks, for example by implementing musician specific exercise programs and increasing health education within higher music education. Other studies examined health education as part of the curriculum for music students (Matei et al., 2018; Matei and Ginsborg, 2022). A study by Perkins et al. (2017) examined how conservatory students experienced health and well-being within their institutions. They used a health promotion framework that focused attention on enablers and barriers to optimal health at three levels: lifestyle, support services and the conservatory environment (Perkins et al., 2017). They identified high work pressure related to rehearsals, lack of support in the music environment and lack of music-specific competence in health services as barriers for the health of music students. Enablers were identified as the value musicians put on optimal health, well-being, and the daily practices to enable this; accessible support services, positive and enjoyable performance experiences, strong relations and communities. Furthermore, Perkins et al. (2017) suggested that the music education institutions’ educational and policy environments should be more supportive and contribute to integrating health-topics in music education (Perkins et al., 2017).

Higher music education in Norway lacks a specific curriculum related to music students’ health, although some of the institutions have short courses, for example the Norwegian Music Academy (Norwegian Academy of Music (NMH), 2023). Examples of themes in the course: The body and the instrument, musicians playing related disorders, sleep and nutrition, however, other countries have developed research based health education courses that have been evaluated (Matei et al., 2018; Matei and Ginsborg, 2022). The Norwegian school of music is closely connected to the ideals of the welfare state and social democracy (Nielsen et al., 2023) and music students in higher education can apply for loans and grants (Lanekassen, 2023). The music education contains both theory and practice and includes music genres like, classical-, folk-, jazz- and rhythmic genres. Instrument teachers have a very central role and are close to the students through their master-apprentice teaching (Fjellman-Wiklund et al., 2002). Previous research on musicians` health in Norway has mostly focused on health related challenges and less on health promoting resources (Trondalen, 2016).

However, a Norwegian study explored what affected the mental health of Norwegian freelance musicians by mapping the experiences of successful freelance musicians from various music genres, related to their particular psychosocial work environment (Vaag et al., 2014). No music students were included in the study, but they used a qualitative method and 12 in-depth interviews were conducted (Vaag et al., 2014). They found that important resources for life as freelance musicians were the individual’s personal resources, such as entrepreneurial skills, flexibility and tolerance for uncertainty (Vaag et al., 2014). Other resources were related to social support and relationships with family, other musicians and the professional network (Vaag et al., 2014). Important contextual challenges for the musicians were related to an uncertain future and finances, difficulties in balancing work and family obligations, as well as massive external pressure (Vaag et al., 2014). A more recent study conducted in Norway by the same authors investigated the prevalence of symptoms, and self-reported disorders of anxiety and depression among 880 music and art students, compared to the general student population (Vaag et al., 2021). A little under half of the music student sample (*n* = 255) were studying performing arts (dance, theater, musical theater, musical performance). The study found higher rates of anxiety and depression in music and art students compared to the general student population in Norway, as well as in the music students compared to art students and highlighted the need for health promotion in Norwegian music education (Vaag et al., 2021). The authors state that the findings should be interpreted with caution because this was a large cross-sectional study using a self-reporting questionnaire. Moreover, the categories of music students included were mixed groups ranging from those doing music teacher training/music therapy to performance artists. The authors encourage more research that looks closely at contextual factors such as high degrees of competition, performance pressure, and demands that may indicate a need to focus on more health promotive and preventive measures within the education and professional music industry in Norway (Vaag et al., 2021).

Trondalen (2016) explored a resource-oriented method of guided imagery and music as a creative resource among 10 musicians and classical music students. The findings indicated that this method could be a creative health resource to foster identity and nurture personal and professional resources. Although this study did not directly address health literacy, Trondalen (2016) suggested that music education should include health promotion. However, the researcher conducted both the intervention and the interviews, which may have biased the results. Although these studies provide some insights in musicians` health there is still limited research on how health promoting resources enable music students in Norway to manage health challenges. Thus, the aim of this study was to explore Norwegian music students’ perceptions and experiences of resources and challenges for health and address the following research question: What health-related challenges do music students in higher music education meet, and what health promoting resources do they need and use to deal with these challenges and promote their health?

## 2. Materials and methods

### 2.1. Theoretical framework

**Figure 1:**
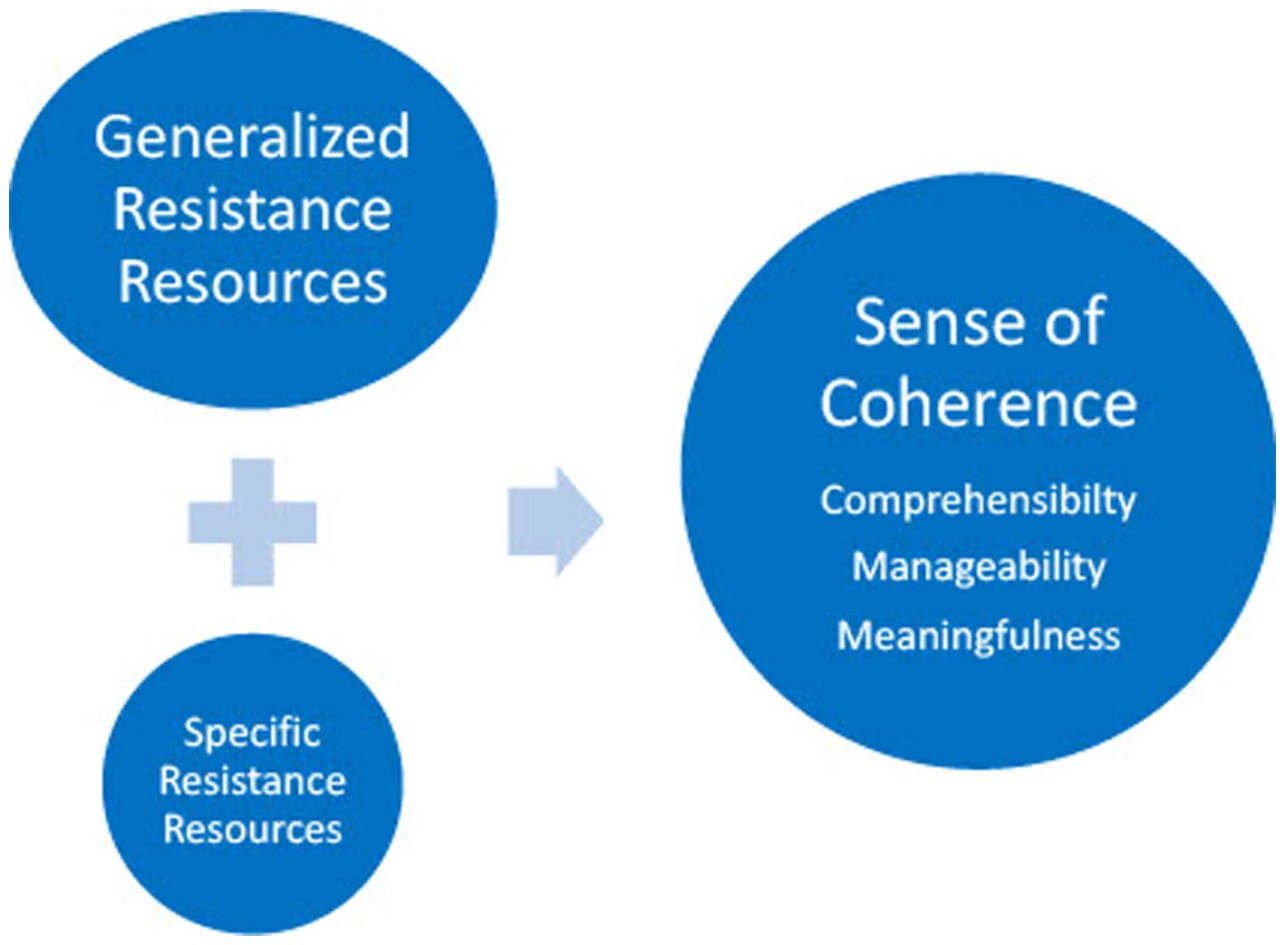
The three key concepts in the Salutogenic model of health, generalized and specific resistance resources and sense of coherence.

Antonovsky (1979) proposed salutogenesis as a theory for health promotion (Antonovsky, 1996). He stated that health and illness were not dichotomous but were placed on either end of the ease dis-ease continuum. He theorized that throughout life we move back and forth along this continuum and that it is important not only to understand what causes disease, but also what builds health, which is the core aim of health promotion. Antonovsky’s (1979) Salutogenic model of health explains how personal, social and environmental resources may be engaged and used to meet challenges and help to build health. Thus, the model provides a useful framework for our study as it includes perspectives on both risk factors and resources for health (Lindström and Eriksson, 2015). The model has three key concepts: (1) Generalized and (2) Specific Resistance Resources and (3) Sense of Coherence (Antonovsky, 1979; Figure 1).

Generalized Resistance Resources are a spectrum of resources, both personal, social and environmental used to meet general challenges in life (Antonovsky, 1979). They include material resources, knowledge and intelligence, real world knowledge and skills, identity and self-knowledge, social support, religion, spirituality or philosophy etc. (Idan et al., 2017). Unlike Generalized Resistance Resources which are developed over the life course and which an individual ‘carries’ with them, Specific Resistance Resources are accessed only to cope with a specific challenge, for example going to the doctor when you are ill (Mittelmark et al., 2017). The Sense of Coherence is a life orientation explaining people’s ability to engage different resources for coping with different types of challenges and consists of three dimensions: comprehensibility, manageability and meaningfulness (Antonovsky, 1987; Mittelmark and Bauer, 2017). Comprehensibility refers to the cognitive understanding of situations and the ability to judge reality. Manageability describes the ability to identify and use the resources we have at our disposal to master challenges. Meaningfulness is the decision that the situation is worth engaging with and contains both emotional and motivational elements, important driving forces for change and action (Antonovsky, 1987).

Saboga-Nunes (2017) constructed a model showing connections between the Salutogenic model of health and health literacy. The connections were measured using the European Health Literacy Survey and the 13-point scale for measuring the Sense of Coherence (Saboga-Nunes, 2017). The results showed that improving health literacy also promoted young people’s Sense of Coherence (Saboga-Nunes, 2017). Comprehensibility was related to understanding of health knowledge. Manageability was related to assessment and competence of health topics and Meaningfulness to understanding and use of digital health information and motivation to be engaged in health-related topics (Saboga-Nunes, 2017). In this study, we have drawn on both Saboga-Nunes’s model, and the Salutogenic model of health to help analyze and interpret our data by focusing predominantly on the sense of coherence. Furthermore we attempt to identify how the concepts that build sense of coherence–comprehensibility, manageability and meaningfulness–are expressed and experienced by the music students to help improve their health literacy and promote their health.

### 2.2. Design and participants

We used a qualitative design and conducted three focus group interviews to explore music students’ understanding, experiences and use of health-related resources in practice and for meeting performance-related health challenges (Creswell and Creswell, 2017). The inclusion criteria were that the participants were current music students and that they understood and spoke Norwegian fluently. The participants were recruited from five higher music education institutions within different regions in Norway. The administrative departments of the educational institutions were contacted via e-mail and provided with information letters and letters of consent. At one institution, one of the music teachers recruited the students. At the other institutions, students received information about the study from their student organizations. Sampling was purposive after they had expressed interest in participating in the study. The focus groups were all conducted in Norwegian and some of the data has been translated into English for this article. During the focus group discussions, we used a semi-structured interview guide with five themes: 1. General questions about musicians and health. 2. Perceptions and experiences of challenges for health. 3. Perceptions and experiences of resources for health. 4. Health literacy within music education including health information and health knowledge. 5. Music culture and health behavior.

Thirteen music students, nine women and four men aged 19–31 years, took part in this study. They were studying classical- (5), folk- (4) and jazz music (3), while one studied rhythmic genres. Five participants were first-year students, seven were in their second-year and one was a fourth-year student. Their instruments were voice, keyboard, wind, string, brass and percussion. The first author (GE) conducted all three focus group interviews together with a co-moderator. The first author and the co-moderator had experience with conducting focus groups together since they had used the method in a previous project. None of the authors had any previous relationship with the participants. The first focus group interview was conducted in October 19, 2020, and included four participants and was conducted in person in a seminar room at one of the universities. These students were defined as a cohort regarding to the COVID-19 protective guidelines, however we still took precautions to sanitize our hands before and after the interview, and to sit 1 meter apart during the interview. The remaining two groups included five and four participants, respectively, and were conducted digitally on Zoom because of the COVID-19 guidelines. One of the groups was conducted on December 17, 2020 and the last group interview was conducted on January 21, 2021. In the first group, the participants knew each other. This made the interaction in this group easier, while in the two digital groups, most of the participants did not know each other or knew only one or two people. We were aware of this during the focus groups and considered it during the analysis as it had the potential to influence their interactions and our findings.

### 2.3. Ethical considerations

Informed consent was obtained from all participants in the study prior to the focus group discussions. The participants were given written information about their right to withdraw from the study at any stage of the process and were provided with information on who to contact if they had any concerns. They were given the transcriptions from their interviews to read through and could give feedback afterwards. The Regional Committee for Medical and Health Research Ethics in Norway, granted ethical approval (REK 170187). Students in higher music education in Norway are a relatively small group, thus efforts were made to maintain anonymity and confidentiality throughout the research process by storing the personal and health information in accordance with recommended guidelines. Data was stored at the University of Bergen digital secure access to research data and infrastructure (SAFE), to ensure secure processing of sensitive personal data. All information was processed without names and national identification numbers or other directly identifying information. At the beginning of each focus group, we discussed the importance of maintaining anonymity and respecting confidentiality among the participants so everyone could speak freely and they were assured that participation was voluntary and they could withdraw consent at any time without any repercussions.

### 2.4. Analyzes

The interviews were recorded and transcribed verbatim and analyzed using thematic network analysis (Attride-Stirling, 2001), with a focus on identifying resources for health. We used the Nvivo 12 Pro (Lumivero, 2017) program to code, systematize and organize the transcripts (Thagaard, 2018). This method consists of six steps: *Step 1:* The text was broken down into segments and text that referred to similar issues was regrouped together to create a coding framework (Attride-Stirling, 2001). This step was conducted inductively by the first author (GE), together with a fellow student during group supervision to improve the rigor by comparing their understandings of the text. The second author read all the transcripts and both the second and the third authors gave feedback during the analysis. After the initial coding, *step 2* was to develop basic themes: these themes say little about the text as a whole on their own and need to be read together with other basic themes to be understood (Attride-Stirling, 2001). Examples of basic themes are “needs for health knowledge” and “opportunities to influence.” In *step 3*, we grouped the basic themes together deductively based on the research questions and the theoretical framework to form organizational themes. Examples of organizational themes were “situational understanding” which relates to Comprehensibility in Sense of Coherence, and “education to support of health” which relates to health literacy and health education. *Step 4* involved identifying and creating the highest level themes in the network, global themes. These themes illuminate the data as a whole and link the individual analysis to the broader societal context (Attride-Stirling, 2001). An example of a global theme is “resources for support of music students` health.” We developed 26 basic, seven organizational and two global themes. Summarizing of the thematic networks was done in *step 5*, and in accordance with Attride-Stirling’s (2001) method, *steps 4 and 5* are encapsulated in the results chapter. S*tep 6, involved* presentation and interpretation of patterns and covered in the discussion section. Table 1 presents the thematic network analyzes with an example of how the basic, organizational and global themes were constructed.

**Table 1:** The six steps in thematic network analysis with an example of the forming of basic, organizational and global theme.

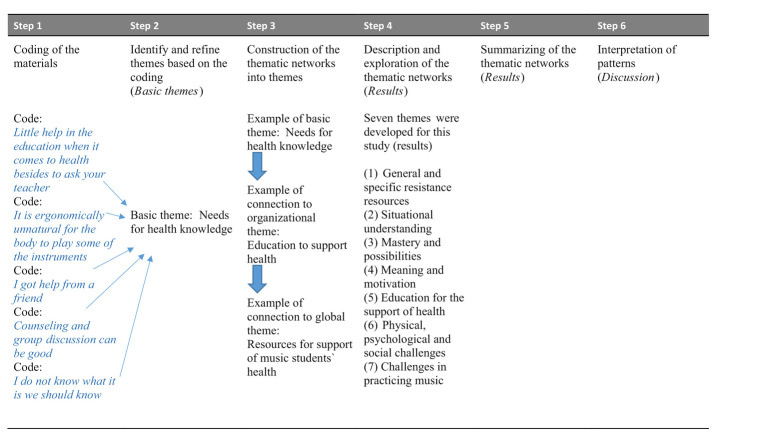

## 3. Results

**Figure 2:**
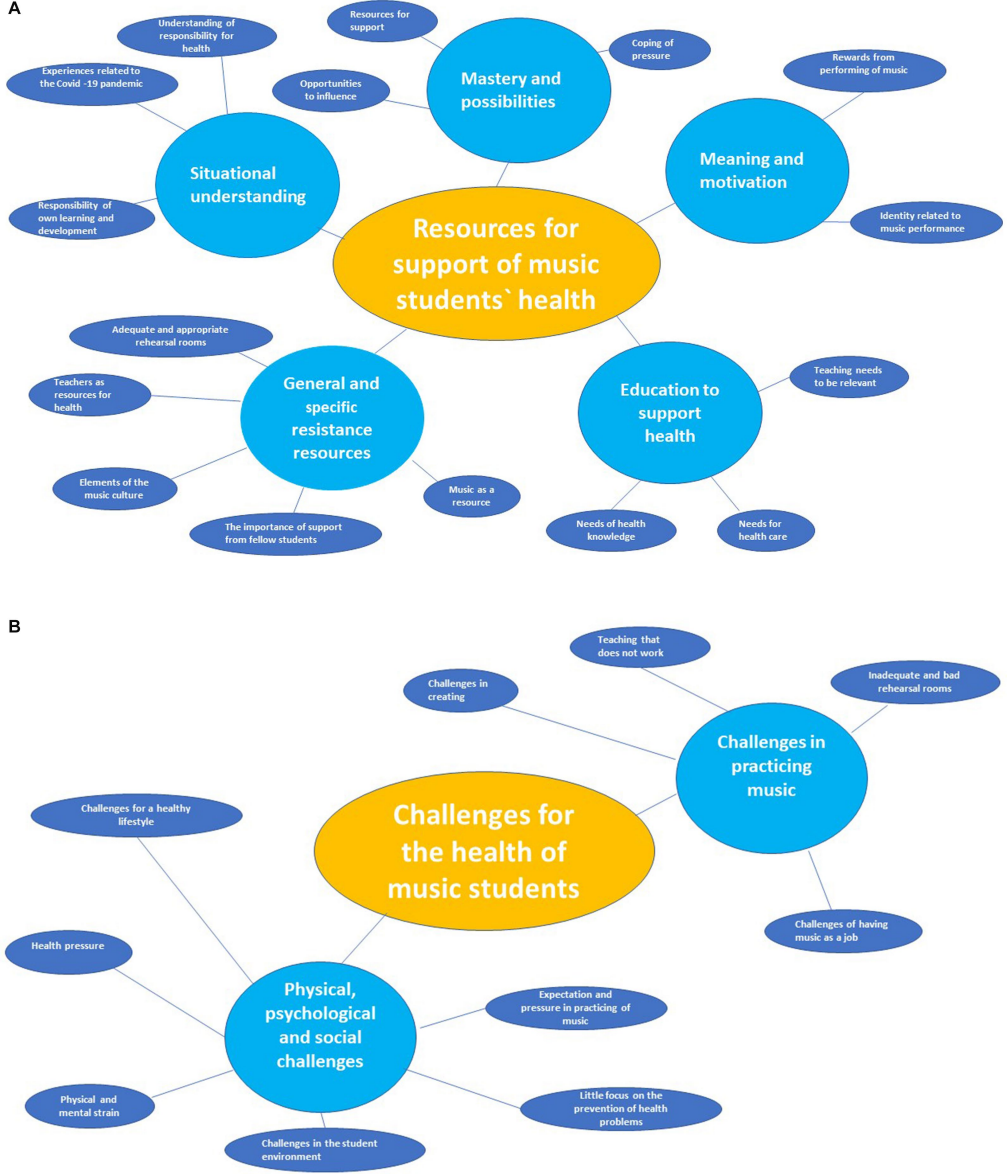
The results of the thematic network consisting of 26 basic, seven organizational and two global themes.

The thematic network consisting of 26 basic, seven organizational and two global themes is shown in Figures 2A,B. The two global themes were: (1) Challenges for the health of music students and (2) Resources for support of music students’ health. Our analysis produced seven organizational themes: (1) General and Specific Resistance Resources, (2) Situational understanding, (3) Mastery and possibilities, (4) Meaning and motivation, (5) Education for the support of health, (6) Physical, psychological and social challenges and (7) Challenges in practicing music.

### 3.1. Challenges for the health of music students

#### 3.1.1. Physical, psychological and social challenges

The participants described health related challenges that we grouped into three categories: physical, psychological and social. Burnout and stress were the psychological challenges most cited. The participants also mentioned physical health challenges like tendonitis and strain injuries, but performance pressure was expressed as the main challenge:

Performance pressure is a mental challenge. I think you can feel lonely. Everyone feels the same loneliness, in that you have a performance pressure or rehearsal pressure or are nervous and constantly living under a pressure to make improvements at every rehearsal. [Student 8].

They were also concerned that maintaining a healthy lifestyle could be an additional pressure to performance pressure. The students` social environment was very competitive, and they mentioned that they tended to talk their achievements down. This was a result of a combination of internal and external expectations increasing a feeling of pressure. They said that it was sometimes difficult to prioritize getting good quality sleep because they sometimes worked late and then it was part of the culture to go out drinking after concerts. Further, some of the participants shared that they had experienced a challenging start to higher music education. They felt that they were expected to put in long hours of practice in order to excel.

It needs to be 40 hours a week, right [Student 1]. The 10 000 hours we really need to be excellent [Student 4].

They reported finding it difficult to adapt to a healthy lifestyle and to include health-promoting practices in their everyday lives as music students in addition to practicing intensively.

It is only when we get an injury we think about our health behavior, because it is only then we discover the health challenges [Student 3].

If they needed treatment or health care, some of the music students had bad experiences with the health care providers’ lack of knowledge about their specific needs and everyday lives as musicians.

They need to understand how our profession actually works without judging, instead of thinking poor musicians, they cannot sort themselves out [Student 3].

An environmental challenge mentioned by the participants was the poor quality of rehearsal rooms. At one of the universities, students had to change rooms every hour due to of the inadequate number of rooms and at another university; the room standards were very poor.

It is a listed building and then you are not allowed to do anything.. or renovate it in any way so almost all the windows have mold on them .. There is almost no soundproofing. You hear everyone rehearsing across the hall when you are in a room [Student 7].

#### 3.1.2. Challenges in practicing music

The participants pointed out that music students lived in their own “bubble of music” and their education did not prepare them for professional life.

I have met musicians that thought that the whole world was interested in their music, but then you suddenly “hit the wall” when you arrive in the real world, because you did not know anything about the real world, you had no idea how to approach other people [Student 8].

Other challenges shared were that performing music required a lot of organization; when students were hired to play at a gig, they feel uncomfortable to ask about payment, and that non-musicians found it difficult to understand that even though they were music students, this was also their professional career.

### 3.2. Resources for support of music students health

#### 3.2.1. General and specific resistance resources

Our findings indicated that music students in higher music education in Norway had several resources that may promote health. Music could be identified here as a Generalized Resistance Resource since they could express feelings through playing and composing it and it was something that was presented as an integral part of the participants’ lives and that promoted their and others health.

You have found a cure that works incredibly well if you use it correctly. When you look back, your darkest moment could be expressed through music, and perhaps be useful for yourself and others [Student 2].

Sharing music with others was also identified as a resource for health, both for the music students and the audience. The sharing of music was expressed as creating a special social community with the audience and removing some of the performance pressure.

To be a part of the wave and the expression you have .., it creates a kind of community and when I see the space, the community that the music creates, it becomes extremely positive [Student 9].

Relationships with fellow students were expressed as important social interactions. A good relationship with fellow students provided important social support.

The most important thing for me is to be taken seriously by people who are in the same situation as me [Student 2].

Teachers on the other hand could be identified as Specific Resistance Resources, as they provided specific support during the study time.

The teachers who rock as educators and are very good at talking to students, if you find them it is very nice [Student 11].

#### 3.2.2. Situational understanding

As data was collected during the COVID-19 pandemic, the participants brought up this topic during the focus group interviews. The participants had heard that musicians in Norway felt financially insecure and were unsure of the future. Recently graduated students in particular seemed to be one of the occupational groups struggling most during this period, however, our participants felt that they had a safety net because of the welfare system in Norway. One participant expressed that the COVID-19 situation had not changed her view of her future. This may be linked to the concept Comprehensibility in Sense of Coherence because it reveals an understanding of her situation during the pandemic.

I do not think corona has changed the thoughts about my own future so much because singers have zero job security anyway. I knew when I started my education that this is an incredibly uncertain future [Student 5].

This may also indicate the importance of Meaningfulness when engaging with a stressor, despite knowing that being a musician is full of uncertainties, the student was still motivated to study music.

#### 3.2.3. Mastery and possibilities

The participants drew on different resources when they met health challenges and found ways to manage their situations. Some of them enjoyed taking part in regular physical activity while some had negative experiences with exercising. However, most of the participants expressed that physical activity was important for promoting good health.

At least I like to think that music students are better at taking care of their physical health than students in general in Norway. I have realized ninety percent of the students exercise, in one way or another, and I think that is because we are aware that the body is half of our instrument [Student 7].

They pointed out the importance of taking responsibility for their own musical development. For example, if the interaction with a teacher did not go well, one had to take the initiative to make a change.

Now I have this teacher, I love every hour and it's absolutely amazing, but the other teachers were not like this one, but they may be good teachers for someone else, as a student you must take responsibility for own health and your own education in a way [Student 8].

From an environmental perspective, the amount and quality of rehearsal rooms was important for practicing music. One student made the room nicer by bringing personal items to the room during practice. Access to adequate rehearsal rooms was expressed as positive.

Good access to rehearsal rooms with a view and daylight is positive and makes the music sound better. If there are large windows, the rehearsal will be better. Strange, but true [Student 8].

Thus, the participants exhibited manageability in different ways, through finding positive solutions to deal with potentially challenging situations. One example was to use the feeling of nervousness before concerts, as a resource. They could actively use the energy experienced from the physical and psychological stress before performing in front of the audience. The students used memories of positive experiences from previous concerts and reminded themselves that playing music was worth the nervousness. Other positive strategies to master performance pressure were to step into a role or to put on an imaginary mask during concerts.

#### 3.2.4. Meaning and motivation

Meaning and motivation are two concepts that are closely linked and that are encompassed under meaningfulness in Sense of Coherence. If one finds an activity meaningful then one is more likely to engage in that activity. The participants in our study expressed high motivation for playing music. Several of them shared that their identity was associated with playing music and they would not give up music no matter how demanding it was.

I stop to reset and take a step back to see my situation from the outside, and the advantages outweigh the disadvantages, since I have a job that I love. There is something new all the time and yes, you are passionate about it [Student 2].

Motivation was important in their daily lives as music students.

It is actually most of all about not giving up on the things you want to achieve, even if you may not know exactly what it is yet, then it will be enough in the end [Student 7].

Another student expressed it like this.

It is large part of my identity. If I do not become a musician, what will I do? [Student 3].

They also said that it was a gift to work with their passion and talked about a great inner drive and love of music. Sharing and playing music was extremely meaningful.

There is no job security in this profession at all, and it is the "sacrifice" you make to keep doing what you love most in the world. Few people get to study and work with their passion. Most people I know have a hobby on the side, that is what they really care about, but they work as an accountant or something, right, you need to have a job. So we are actually very privileged [Student 5].

#### 3.2.5. Education to support health

The participants expressed that increasing health-related knowledge in Norwegian music education would be a resource for music students. They pointed out the need for health education that is relevant for musicians to be integrated when they are taught their instrument. They stated that the importance of regular physical activity also needed to be connected to the importance of practicing their instruments, and that it would be useful to get feedback on how to move correctly when playing their instruments. Other aspects they brought up were the importance of being social with other students and how socializing positively affected their mental health. They also mentioned the need to increase the health-related knowledge of music teachers.

Your teacher may say you have to practice in a healthy way and take many breaks. How do you know when you do it right, what are enough breaks? There is very little information and few resources. I think almost all music students are struggling with different health issues [Student 7].

The students considered career guidance and courses on how to cope with stressors and performance pressure as most urgent. Furthermore, they expressed uncertainty about what health information and knowledge they needed as music students.

I do not know what we need to know [Student 1].

## 4. Discussion

Our findings revealed several resources that promoted the music students’ health: *personal resources* included understanding of reality, using coping strategies, motivation and regular physical activity. *Social resources* involved understanding the importance of reaching out for social support from peers and teachers and synergy created between themselves and the audience through sharing of music. *Environmental resources* were linked to access to good rehearsal rooms or finding ways to improve the ambience in rehearsal rooms. The main health challenges were performance pressure and difficulty integrating good health habits. Finally, the students expressed a need to increase their competence in health literacy and to implement health education in the Norwegian music education. To our knowledge, this is the first study in Norway exploring this topic across a broad selection of music genres.

### 4.1. Challenges for the health of music students

Music students may be particularly vulnerable when they start their higher music education. Our participants discussed how this early period in their education was challenging and that they felt insecure about themselves and their music. Several studies have made similar observations (Spahn et al., 2017; Pecen et al., 2018; Alessandri et al., 2020). Pecen et al. (2018) discuss the need for psychological preparation and support especially for the new students. This is consistent with our participants, who expressed that they wanted a preparatory course to learn to master performance pressure early in their education. A course like this may serve as a Specific Resistance Resource to meet the stressor of performance pressure. It may help to manage the stressor but is not likely to prevent avoidance of the stressor or help to redefine it as not being a stressor (Mittelmark and Bauer, 2017). However, learning to manage stressors may promote health and stressors can be Salutogenic if they are understandable, manageable and meaningful (Antonovsky, 1979). The lack of a national curriculum for health-related teaching in higher music education in Norway is consistent with previous research from the United Kingdom (Perkins et al., 2017). In Matei and Ginsborg’s (2022) study, a health education course for music students was positively evaluated, but the students called for less theoretical and more practical education. Sufficient health education and health literacy may contribute to promoting health in music students.

The COVID-19 pandemic increased social challenges and the music students had heard from professional musicians that the pandemic increased their financial insecurity and uncertainty about the future as many musicians’ livelihoods also depend on live performances. These uncertainties are be fundamental challenges for music students, and the COVID-19 pandemic may have increased these fears (Rosset et al., 2021). A recently published study from Germany on music students’ challenges and health during the COVID-19 pandemic, revealed increased fear for the future in 19% of the students (Rosset et al., 2021). Worrying about the future due to financial uncertainty seems to be a stress factor at the group level for music students (Antonovsky, 1979). On the other hand, perceptions of stressors are subjective and contextual (Antonovsky, 1979). Our participants experienced Norway as a country with strong financial security, giving them a safety net in contrast to their peers in other countries where the same safety net with financial support is absent. Living in an environment that provides financial stability through supportive social security structures may strengthen the Norwegian music students’ sense of coherence as they experience their financial situation as comprehensive, manageable and meaningful. In our study, no foreign students were included since an inclusion criterion was that the participants had to understand and speak Norwegian fluently, and differences between students from different countries based on the financial situations were therefore not be explored.

The physical environment is also an important aspect of health promotion and seems to be underestimated in music education. Several of our participants experienced that the quality of the rehearsal rooms had a negative impact on their health and on the quality of their rehearsals. These students spent many hours in small rooms without windows and with poor ventilation. This experience has not been mentioned in previous studies. Even though educational institutions are responsible to ensure that environmental factors promote students’ health and safety, challenges related to rehearsal rooms seem underestimated (Kreutz et al., 2009). The most important aspects for our participants regarding satisfactory rehearsal rooms were access to daylight and large windows. Rooms with these features were experienced as positive, and the students noted that the rooms improved the quality of sound during practice. Alternatively, some participants took the initiative to improve poor quality rehearsal rooms by bringing in personal items. This way of dealing with the challenges associated with practice rooms highlights students’ ability to understand a situation and assess it as manageable and to draw on coping resources that reduce the effect of a stressor (Antonovsky, 1987). However, it might also indicate a lack of support from or engagement with their educational institutions, since our participants did not talk about raising these issues with the student union or departmental administration to demand better facilities. It may also show a need to increase health literacy among music students, so they are aware of their rights to healthy learning environments. Moreover, other approaches such as the need for supportive health policies and increased awareness of the social determinants of health are warranted (Green et al., 2019). The social determinants of health are related to general socioeconomic, cultural and environmental conditions and need to be taken into account since they are part of the core principles of health promotion (Green et al., 2019).

Our participants, regardless of music genre, had experienced inadequate health care because the healthcare providers did not have enough knowledge or understanding of the participants’ challenges related to playing instruments. They expressed examples of health care providers who did not understand how the music profession worked and were judgmental. There seems to be less research exploring musician’s health from other genres than the classical genre (Wilson et al., 2014). More studies need to explore and increase knowledge and experiences of music students who perform within different genres (Perkins et al., 2017). Further, there is limited research on folk musicians compared to classical musicians (Wilson et al., 2014). A study of Irish folk musicians showed a lack of competence in the health service with regard to their specific health problems (Wilson et al., 2014). Moreover, a Danish study showed that symphony orchestras lacked support from a competent health care system (Paarup et al., 2011). This might explain the findings from Langvik et al. (2019) where Norwegian musicians used alternative therapies more often than the general population in Norway (Langvik et al., 2019). Moreover health related knowledge and competence are not sufficient or available, musicians and music students may turn to negative coping strategies such as alcohol (Kenny et al., 2014; Vaag et al., 2016; Pecen et al., 2018). In our study, the participants talked about how alcohol could be a part of the lifestyle after concerts late in the evening, but they did not point to it as a coping strategy, and they did not mention any other stimulants.

When you do not know what you need to know, you have less opportunity to be in control of and promote your own health (Green et al., 2019). Research indicates that health education and health literacy provide music students with greater opportunities to influence their health (Matei et al., 2018). According to Matei et al. (2018), health literacy within a health-promoting framework might support music students to engage and be able to influence their conditions. Health literacy is important both in terms of identifying and in terms of critically assessing health information from different sources (HOD, 2019). Music education institutions should support health promotion policy, facilitate supportive environments, as well as provide competent health service providers (Matei et al., 2018). From this perspective, we can advocate for health education and health literacy as a right for music students (Okan et al., 2019).

### 4.2. Resources for support of music students health

The social aspects of physical activity were of great interest for the participants in our study. Physical activity was a good diversion from the “bubble of music,” and an opportunity to be sociable and active with other students. In the Salutogenic model of health, social networks and relationships are highlighted as important Generalized Resistance Resources (Antonovsky, 1979). To develop health literacy, promoting healthy habits can occur through interactions between individuals and their social environment (Sorensen et al., 2012). Findings from other studies have shown that social support is an important resource for musicians and music students (Vaag et al., 2014; Philippe et al., 2020). Vaag et al. (2014) revealed that strong social relationships and support contributed to a high Sense of Coherence for freelance musicians in Norway. Further, findings from a Swiss study indicated that social support was important for both the high performance of music students, and promoting well-being (Philippe et al., 2019). Music students who mostly worked and performed alone were lonelier and had a greater need for social contact than those who practiced and performed in groups (Philippe et al., 2019). The Salutogenic model of health points to social relations and social support as crucial for health promotion.

A perspective that has received less attention in research is the synergy created between the music students and the audience through performance. Our participants expressed this as an extremely positive experience and one described it as ‘magical’. Ascenso et al. (2018) highlighted the synergy created when music is shared with the audience as an important psychological resource for musicians. Their study also showed that a sense of community is created when musicians perform together (Ascenso et al., 2018). Our participants expressed that feeling nervous before concerts could be used actively as energy and be a resource. This is in line with how a stressor may be Salutogenic (Antonovsky, 1987). They used the previous positive experiences to support themselves and focused on their passion for music. It could be helpful to step into a role or to put on an imaginary mask during concerts. The participants expressed that sharing and playing music was extremely meaningful. According to the Salutogenic model of health, meaningfulness is central for drive and motivation, and is the key concept in the development of a Sense of Coherence. The participants’ identity was associated with playing music, and they declared that they would never give it up no matter how demanding it was. This is consistent with the results from Ascenso et al. (2018) that indicated being a musician as the most positive psychological resource. Health-related topics need to be integrated with music-related topics in the education to be meaningful for music students. If they can identify the meaningfulness of engaging with health-related topics and link that to their identity as musicians, it might increase motivation, which is an important driving force for engaging with health literacy and health education (Green et al., 2019).

Health literacy and health education are important health promoting resources that can contribute to support good decisions about health in music students’ everyday lives (Kickbusch et al., 2005). Health literacy develops during interaction between the individual and their social and structural environments. However, research shows that institutions educating musicians prioritize neither health literacy nor health education. Findings from our study indicated that teachers from music education institutions provided students with little support and information about health behaviors directly related to practice and performance. This is consistent with a qualitative study from the United Kingdom investigating factors that promoted and inhibited optimal health and well-being in music students (Perkins et al., 2017). The results showed that there were several barriers for health promoting values and lifestyles (Perkins et al., 2017). It is important to engage institutions to promote health-related knowledge, discuss topics relevant to health and foster motivation for healthy behaviors in order to increase health literacy and health education among music students. Health-related topics should be included in curricula and institutional policies.

Our findings imply that efforts to promote an increase in the levels of physical activity among music students should highlight the positive social aspects of physical activity. Furthermore, it is important to make physical activity visible as a resource for both music-specific challenges and for promoting mental health. In our study, the participants recommended more focus on the importance of implementing physical exercise in their education. This is in line with Araújo et al.’s (2020) findings that highlighted the importance of teaching music students about how the body works, as well as specific training related to instruments and genres. According to Araújo et al. (2017), health education and health literacy do not seem to be integrated into music education today. To increase health literacy among music students, it has to be integrated as a topic in the educational programs, otherwise it may not be meaningful for the music students to engage with health-related topics, and they may not be motivated.

## 5. Limitations

The study sample was small, but the number of focus groups seemed adequate to answer the research questions as no new topics emerged in the final focus group (Malterud et al., 2016). However, there might have been information we missed by having this sample size. Other participants may have uncovered new topics. The first author is a health professional, which may have biased the interview questions and the themes emerging from the focus groups, but the interview guide was pilot tested to ensure the questions were clear and easy to understand. The second author works within health promotion and provided feedback during the study together with a group of colleagues, who were working on other health promotion projects. The advantage of digital groups was that we could gather students from five different music schools in different regions of Norway, and thereby gain perspectives from different educational institutions, which would have been difficult to achieve in person. However, it is important to note that the use of digital methods may also lead to challenges and limitations, both methodologically and ethically (Gill and Baillie, 2018). It was difficult to ensure that the participants did not have other people around who could listen to the interview, and we had to actively address this problem and pause an interview once when we heard other people talking in the background. Another limitation of only using focus groups is that some students may have spoken more freely in one-to-one interviews than in a group. The students were in different years of their studies and some of them might have spoken more freely in groups with students from the same study year.

## 6. Conclusion

Health is important in everyday life for music students. The findings from this study with Norwegian music students revealed several resources on a personal-, relational-, social and environmental level that promoted the students’ health. On a personal level, the resources included situational understanding, using coping strategies, motivation and regular physical activity. On a relational and social level, resources were related to understanding of the importance of reaching out for social support from peers and teachers, and synergy created between themselves and the audience through sharing of music. At an environmental level, the resources were linked to access to good rehearsal rooms or solutions to improve the ambience in the existing rehearsal rooms. The students experienced health challenges as performance pressure and difficulty integrating healthy habits into their daily study lives. They expressed a need for health education and health literacy to be integrated into their education to increase health promoting routines during practice and performance. The findings may indicate that there is a need to develop national strategies and policies to increase health literacy in Norwegian music education. Further research is needed to implement and increase health promotion within the music institutions in Norway. By using a Salutogenic perspective, music education can move in a healthy direction with help from the broad repertoire of resources from the music students.

## Data availability statement

The raw data supporting the conclusions of this article will be made available by the authors, without undue reservation.

## Ethics statement

The studies involving humans were approved by The Regional Committee for Medical and Health Research Ethics in Norway, granted ethical approval (REK 170187). The studies were conducted in accordance with the local legislation and institutional requirements. The participants provided their written informed consent to participate in this study.

## Author contributions

GE, FO, and BF contributed to the design of the study. GE conducted all three focus group interviews together with a co-moderator and wrote the first draft of the manuscript. FO and BF contributed to the analysis and they contributed to the overall writing and revision of the manuscript. All authors contributed to the article and approved the submitted version.

## Funding

This work was supported by a scholarship from The Norwegian Fund for Post-Graduate Training in Physiotherapy (Grant number, 160181, GE).

## Conflict of interest

The authors declare that the research was conducted in the absence of any commercial or financial relationships that could be construed as a potential conflict of interest.

## Publisher’s note

All claims expressed in this article are solely those of the authors and do not necessarily represent those of their affiliated organizations, or those of the publisher, the editors and the reviewers. Any product that may be evaluated in this article, or claim that may be made by its manufacturer, is not guaranteed or endorsed by the publisher.
